# Impact of chest wall motion caused by respiration in adjuvant radiotherapy for postoperative breast cancer patients

**DOI:** 10.1186/s40064-016-1831-3

**Published:** 2016-02-24

**Authors:** C. Lowanichkiattikul, M. Dhanachai, C. Sitathanee, S. Khachonkham, P. Khaothong

**Affiliations:** Faculty of Medicine, Ramathibodi Hospital, Mahidol University, 270 Rama Road VI, Rachathevi, Bangkok, 10400 Thailand

**Keywords:** Breast cancer, Adjuvant radiotherapy, Respiration, Chest wall motion

## Abstract

To determine the chest wall movement of each patient during deep inspiratory breath hold (DIBH) and expiratory breath hold (EBH) in postoperative breast cancer patients. Postoperative breast cancer patients who underwent CT simulation for 3D radiotherapy treatment planning during December 2012 to November 2013 were included. Before scanning the radio-opaque wire was placed on the surface for breast and chest wall visualization on CT images, then the patient underwent three phases of CT scanning (free breathing, DIBH, and EBH, respectively). The distances of chest wall motion at five reference points were calculated using the treatment planning system. 38 breast cancer patients who underwent surgery were included. Median age was 48.5 (28–85) years. Median BMI was 23.4 (16.6–38.3) kg/m^2^. Median lung volume was 3160.5 (1830.8–4754.0) cm^3^. Median Haller index was 2.43 (1.92–3.56). Median chest wall movement was wider in anteroposterior (A–P, 4.2–5.4 mm) than superoinferior (S–I, 2.5–2.6 mm) and mediolateral (M–L, 0.6–1.1 mm) dimension in all five measured points. There was no significant effect of the type of surgery, BMI, lung volume, and the Haller index on the distances of chest wall movement. Additional margins of 7, 5, and 2 mm to the A–P, S–I, and M–L dimension should adequately cover the extreme chest wall movement in 95 % of the patients. This study showed that the maximal movement of the chest wall during DIBH and EBH was greatest in the A–P axis followed by the S–I axis, while the M–L axis was minimally affected by respiration.

## Background

Breast cancer is the most common cancer in women (Attasara and Buasom 2011; Siegel et al. 2012). It is known that postoperative breast irradiation can improve local control in early-stage breast cancer (Fisher et al. 2002). According to the anatomy of the breast that lies on the anterior chest wall, intra-fraction movement of the clinical target volume (CTV) can occur due to respiration during treatment delivery. Large treatment margins added to cover the movement may subsequently cause a substantial volume of normal tissue exposed to radiation resulting in increased risk of treatment-related toxicity (Korreman et al. 2006).

It has been assumed that breast motion from breathing during standard whole breast RT does not significantly affect the dose distribution within the breast tissue. In one study the baseline average movement during normal breathing was <2 mm in all dimensions (Chopra et al. 2006). However, in the extreme phase of respiration like DIBH the distance of chest wall movement can be increased up to about 12.6 mm (Pedersen et al. 2004) According to the European Organization for Research and Treatment of Cancer (EORTC) survey of clinical practice in Europe, there was still variation in an additional treatment margin with the mean value of around 7.5 mm (Hans and Coen 2013).

Lung vital capacity has been shown to strongly correlate with the chest wall movement (Cala et al. 1996). Chest wall deformity also affects the chest wall motion in patients with pectus excavatum, with the motion decreasing markedly at the level of deformity (Redlinger et al. 2011). The severity of deformity could be graded by Haller index, which was the ratio of the chest wall width and depth. Increasing Haller index indicated more severe deformity.

This study was planned to evaluate the movement distances of breast or chest wall during DIBH and EBH and also to evaluate factors affecting chest wall movement during respiration.

## Methods

### Patients

Consecutive postoperative breast cancer patients, planned for CT simulation for adjuvant radiotherapy in Ramathibodi Hospital from December 2012 to November 2013, were included in the study. Prospective data collection was performed. All patients provided written informed consent, and the study was approved by the institutional ethics committee and funded by the Faculty of Medicine, Ramathibodi Hospital, Mahidol University.

### CT simulation

The patients lied on the breast board and were instructed to hold their breath for 20 s for both DIBH and EBH. Patients were trained and scanned in the supine treatment position with the ipsilateral arm lifted above the head and the sternum in a horizontal position. Scanning included a standard free-breathing (FB) scan, followed by DIBH and EBH. The DIBH and EBH scans started immediately after the patients’ chest stabilized at the maximum height and were finished within the 20-s period to maintain breath-holding position of the patients. During scanning, the patients were visualized by the researcher to ensure that they were in the intended breath-holding position. If that was not the case then re-scanning was performed. The area for FB scan included the entire neck and chest wall, whereas for the DIBH and EBH it included only the breast plus 2 cm margin (Korreman et al. 2006; Korreman et al. 2005). The DIBH and EBH scan used the same table speed and slice thickness to cover the entire target area within one single breath-holding cycle. For this reason we decreased the milliampere-seconds (mA s) to both shorten the scan time and to reduce radiation exposure. This caused the reduction of the image quality but it was still good enough to see the radio-opaque markers. Table 1 showed details of the imaging protocol.

**Table 1:** Details of the imaging protocol

Series	Range	mA s	Kv	Table speed (mm/s)	Duration (s)	Slice thickness (mm)
FB	Neck, chest	250	120	5	60	2.5
DIBH/EBH	Chest	80	120	5	20	2.5

**Fig. 1:**
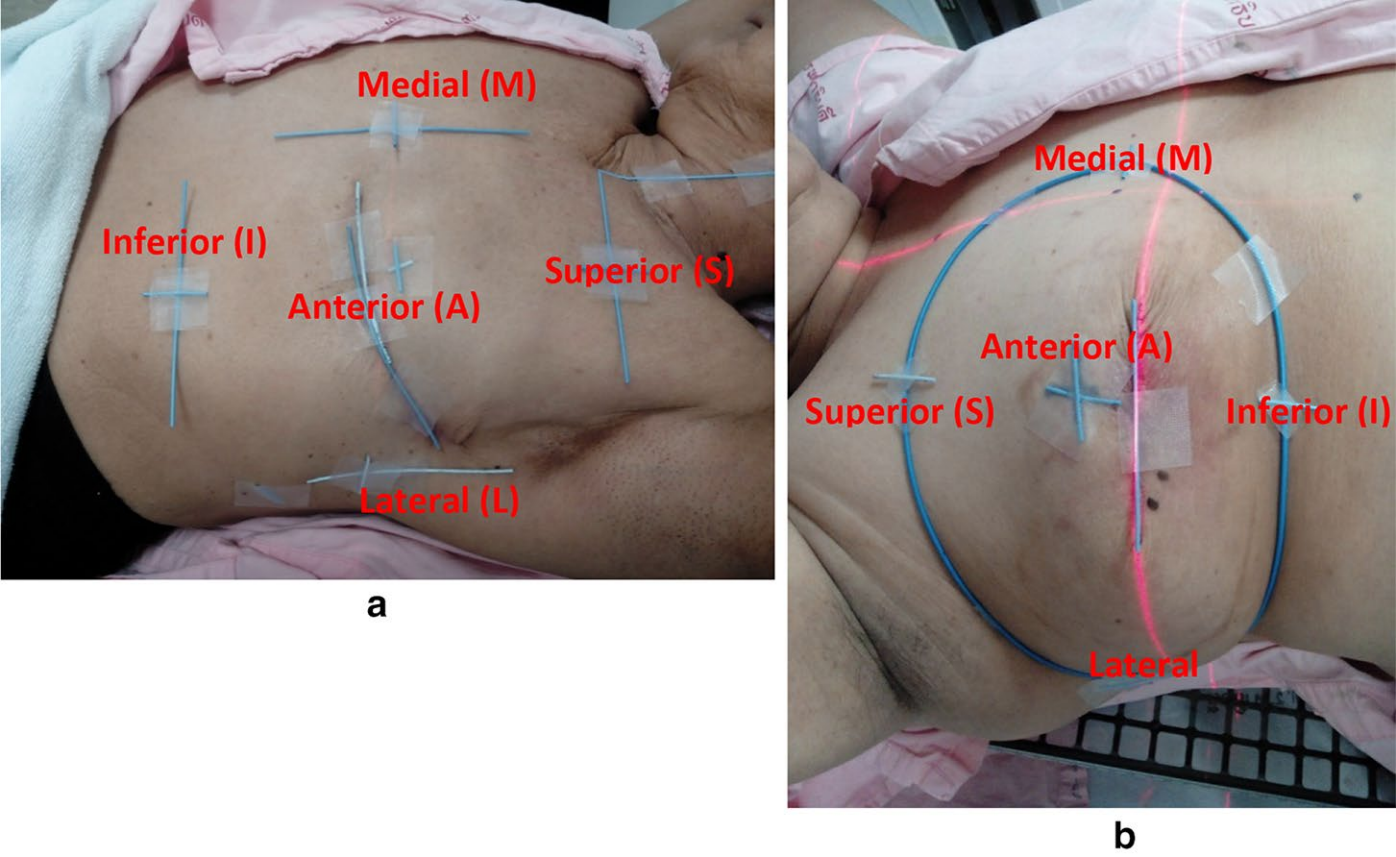
Placing the radio-opaque wires. **a** Mastectomy. **b** Breast conserving surgery

Orthogonal room lasers were used to position the patient and to place ink marks with radio-opaque markers on the skin surface. These markers were used to verify that the patient did not shift relative to the angle board and the CT table between the scan series (Pedersen et al. 2004; Lu et al. 2000). For postmastectomy patients the radio-opaque wires were placed by the anatomical landmark to outline the CTV, superiorly at the level just below the clavicular head, inferiorly at 2 cm below the infra-mammary fold, medially at midline, and laterally at mid-axillary line, respectively. In patients who underwent breast-conserving surgery (BCS), the radio-opaque wires were placed along the breast contour. When the patients were positioned to the center of the CTV, the points where laser intersected with the CTV border (S for superior, I for inferior, M for medial, L for lateral, and A for anterior at the CTV center) were used as the reference points to measure the chest wall movement. Figure 1 showed how the wires were placed in both groups of patients and the reference points as mentioned.

### Measurement of chest wall movement

Data was collected using the Eclipse^®^ planning system (Varian Medical Systems, Inc, California, USA). The translational movement of the reference points (S, I, M, L, and A) was recorded in all three dimensions, namely S–I, A–P, and M–L. The maximal movement in each direction of the reference points was calculated from the differences between the markers position during DIBH and EBH. Also the movement of the reference points during DIBH and EBH was compared to the FB position.

### Factors affecting chest wall movement

Four factors were explored to find the correlation with chest wall movement including the surgical procedure (mastectomy vs BCS), the body mass index (BMI), the total lung volume during DIBH (lung volume_DIBH_) and the Haller index.

BMI was calculated using the formula:

$$BMI = \frac{{body\;weight\; \left( {\text{kg}} \right)}}{{height^{2} \;\left( {{\text{m}}^{2} } \right)}}$$

Instead of the lung vital capacity, this study aimed to explore the correlation between chest wall movement and the total bilateral lung volume during DIBH. The volume was estimated using auto-contouring tools of the Eclipse^®^ planning system.

The Haller index was used to grade the severity of chest wall deformity and was the ratio of the maximal width of chest wall divided by the minimal distance between the posterior sternum and anterior vertebral body at the same level. In this study, the index was measured in DIBH images (Redlinger et al. 2011).

### Statistical data analysis

Mean, standard deviation (SD), median and range of the translational movement in each axis for each reference point was calculated. Spearman’s rank correlation coefficient was used to describe the correlation between BMI, lung volume_DIBH_, Haller index and the chest wall movement. Mann–Whitney U test was used to compare the difference of chest wall movement between the mastectomy and BCS group.

## Results

### Patients’ characteristics

38 patients were enrolled in the study, 21 had left-sided breast cancer. Median age was 48.5 (28–85) years. 27 patients underwent mastectomy and 11 had BCS. Median BMI was 23.4 (16.6–38.3) kg/m^2^. Median lung volume_DIBH_ was 3160.5 (1830.8–4754.0) cm^3^. Median Haller index was 2.4 (1.9–3.6). The summary of the patients’ characteristics was shown in Table 2.

**Table 2:** Patients’ characteristics

	Number (%)	Median (range)
Age (years)	38 (100)	48.5 (28–85)
BMI (kg/m^2^)	38 (100)	23.4 (16.6–38.3)
PS		
ECOG 0	27 (71.1)	
ECOG 1	11 (28.9)	
Side		
Left	21 (55.3)	
Right	17 (44.7)	
Staging		
In situ	7 (18.4)	
I	12 (31.6)	
II	9 (23.7)	
III	10 (26.3)	
Procedure		
Mastectomy	11 (28.9)	
BCS	27 (71.1)	
Haller’s index	38 (100)	2.4 (1.9–3.6)
Lung volume (cm^3^)	38 (100)	3160.5 (1830.8–4754.0)

### Chest wall movement

Table 3 summarized the distances of the maximal chest wall movement in three axes of each marker. 95 % coverage referred to the distance of chest wall movement of 95 % of the patients. The median chest wall movement in the A–P axis was more pronounced than in the other axes in all five measured points, 4.2–5.4 mm. For the S–I axis the distance of movement was about 2.5 mm. On the contrary the movement in the M–L axis is less, 0.6–1.1 mm. The margins that would cover the chest wall movement in 95 % of the patients were 4.0–5.1 mm for the S–I, 1.0–2.0 mm for the M–L, and 5.5–7.1 mm for the A–P dimension.

**Table 3:** The maximal chest wall movement

	Mean	Median	95 % coverage
	*Point of intersection: superior (mm)*		
S–I	3.3 ± 2.8	2.5 (0.0–10.0)	4.2
M–L	1.5 ± 1.5	1.1 (0.0–6.9)	1.9
A–P	5.7 ± 4.3	5.4 (0.0–20.0)	7.1
	*Point of intersection: inferior (mm)*		
S–I	3.0 ± 2.9	2.5 (0.0–10.0)	4.0
M–L	1.5 ± 1.5	1.1 (0.0–5.8)	2.0
A–P	5.8 ± 4.0	5.1 (0.2–16.2)	7.1
	*Point of intersection: medial (mm)*		
S–I	3.9 ± 3.4	2.6 (0.0–12.5)	5.1
M–L	0.8 ± 0.7	0.6 (0.0–3.5)	1.0
A–P	5.3 ± 3.8	4.3 (0.0–13.1)	6.6
	*Point of intersection: lateral (mm)*		
S–I	3.0 ± 3.2	2.5 (0.0–12.5)	4.0
M–L	1.2 ± 1.4	0.9 (0.0–7.1)	1.6
A–P	4.5 ± 3.2	4.2 (0.0–12.0)	5.5
	*Point of intersection: anterior (mm)*		
S–I	3.6 ± 2.6	2.5 (0.0–10.0)	4.4
M–L	1.3 ± 1.4	1.0 (0.0–7.7)	1.7
A–P	5.7 ± 4.2	5.1 (0.4–14.0)	7.1

Table 4 showed the distances of the chest wall movement during DIBH and EBH comparing to the chest wall position in the FB CT images. Movement in the posterior, superior and lateral direction was given the “+” sign. The distances of chest wall movement were greater during DIBH than EBH comparing to the FB scans in all dimensions of almost all the measured points. During DIBH, the chest wall moved more anteriorly (5.9–7.0 mm) and superiorly (2.5–5.0 mm) than during FB. However, it moved <1.0 mm in the M–L dimension (−0.7 to 0.3 mm). During EBH compared to FB, the distances of movement were less. The chest wall moved 1.1–2.0 mm anteriorly, 0.0–1.2 mm superiorly and 0.1–0.3 mm mediolaterally.

**Table 4:** Distance of chest wall movement during DIBH and EBH comparing to the FB scan

	Median distances (mm)					
	A (−) P (+)		I (−) S (+)		M (−) L (+)	
	DIBH	EBH	DIBH	EBH	DIBH	EBH
S	−6.2 (−21.3 to 0.4)	−1.3 (−14.2 to 6.4)	5.0 (−2.5 to 10.0)	0.0 (−5.0 to 10.0)	0.3 (−2.5 to 8.5)	0.1 (−4.9 to 7.3)
I	−6.1 (−21.2 to 1.5)	−1.1 (−17.1 to 5.8)	2.5 (−2.5 to 10.0)	0.0 (−5.0 to 12.5)	−0.7 (−19.3 to 3.2)	−0.3 (−19.9 to 3.8)
M	−5.9 (−15.4 to 0.0)	−1.2 (−14.5 to 7.8)	5.0 (−12.5 to 12.5)	1.2 (−12.5 to 12.5)	0.0 (−3.5 to 1.8)	−0.3 (−5.3 to 2.4)
L	−6.7 (−11.7 to 3.3)	−2.0 (−15.7 to 4.4)	2.5 (−12.5 to 25)	0.0 (−12.5 to 30.0)	−0.3 (−5.8 to 3.8)	−0.1 (−9.4 to 4.0)
A	−7.0 (−16.5 to 0.8)	−1.5 (−16.4 to 8.9)	2.5 (−2.5 to 12.5)	0.0 (−5.0 to 12.5)	−0.4 (−9.6 to 6.2)	−0.1 (−10.2 to 5.2)

### Factors affecting chest wall movement

Different surgical procedure of the breast affected the shape of the chest wall, however this study showed no statistical significant differences in chest wall movement after mastectomy or BCS, except movement in S–I axis of the lateral marker, which showed more superior movement in BCS as 2.5 mm compared with 0.0 mm (p = 0.041).

For the other factors, data were analyzed to find the correlation between chest wall movement in each axis with BMI, lung volume_DIBH_ and Haller index (HI). BMI and Haller index both showed no significant correlation with the chest wall movement. Comparing between normal BMI and obese patients, although the distances of chest wall movement in A–P direction of the patients with normal BMI were greater than in obese patients (4.7–6.5 mm compared to 2.6–4.3 mm), the differences were not statistically significant. Movement in the other directions was almost the same in both groups of patients.

There was weak to moderate correlation between lung volume_DIBH_ and chest wall movement of only some measured points. In S–I axis, only I marker show significant moderate correlation with r_s_ = 0.405. In A–P axis, I, L, and A marker had r_s_ = 0.390, 0.486 and 0.361, respectively. When lung volume_DIBH_ was categorized into two groups, the patients with larger lung volume_DIBH_ seemed to have more chest wall movement in A–P direction, 5.4–6.7 mm compared to 2.2–4.6 mm in patients with lung volume ≤3000 cc. However, there were no statistical significant differences between the two groups in all other directions of chest wall movement.

## Discussion

Many methods were used to evaluate the motion of the chest wall in breast cancer patients. This study showed how the respiration affected the chest wall movement by using the markers and conventional CT simulation. The comparison of chest wall movement reported from different studies is shown in Table 5. Our study confirmed that the movement in A–P and S–I direction was wider than in M–L axis. The reason why overall chest wall movement in this study seemed to be larger than the previous studies using 4DCT or tracking technique might be the DIBH technique used to demonstrate the extreme phase of respiration. Comparing with the study reported by Chopra et al. which used the similar DIBH technique, the distance of chest wall movement seemed to be in the same magnitude except the movement in the S–I axis which was less in this study. This could be due to the position of the patients during CT scan using the breast board in the patient set up whereas in the previous study the chest wall movement was measured in the supine position without the breast board.

**Table 5:** Chest wall motion reported in different studies

	N	Method	Result (mm)		
			S–I	M–L	A–P
Pedersen et al. (2004)	16	DIBH	–	–	12.6 (8.0–20.0)
Chopra et al. (2006)	5	DIBH	5.5 (1.4–8.2)	2.0 (1.0–3.4)	4.8 (1.7–9)
		FB	1.94 (0.7–4.5)	1.07 (0.6–1.4)	1.86 (0.6–4.9)
Kinoshita et al. (2008)	17	Tracking	1.3 ± 0.5	1.0 ± 0.6	2.6 ± 1.4
Richter et al. (2009)	10	4DCT	–	–	1.8 (0.2–3.8)
		EPID			1.5 (0.8–2.2)
Wang et al. (2013)	17	4DCT	1.38 ± 0.85	1.03 ± 0.48	0.95 ± 0.36
This study	38	DIBH			
Superior			2.5 (0.0–10.0)	1.1 (0.0–6.9)	5.4 (0.0–20.0)
Inferior			2.5 (0.0–10.0)	1.1 (0.0–5.8)	5.1 (0.2–16.2)
Medial			2.6 (0.0–12.5)	0.6 (0.0–3.5)	4.3 (0.0–13.1)
Lateral			2.5 (0.0–12.5)	0.9 (0.0–7.1)	4.2 (0.0–12.0)
Anterior			2.5 (0.0–10.0)	1.0 (0.0–7.7)	5.1 (0.4–14.0)

One reason that might explain why there was no strong correlation between different factors and the chest wall movement in this study was the relatively small number of patients with quite homogeneous baseline characteristics. Most patients were not obese with the BMI of 23.4 kg/m^2^ and there was only one patient with the pectus excavatum. There seemed to be some relationship between the larger lung volume_DIBH_ and the more movement of the inferior, lateral, and the anterior measured points, which in some part might be explained by the more lung volume change in the lower lung region caused by the diaphragm reflecting in different movement of different part of the chest wall.

Naturally the chest wall moves unevenly during respiration, so applying the uniform margin for all directions in chest wall irradiation might result in over treating the normal tissue or under coverage of the CTV. Considering the distances of movement measured in different phases of breathing in this study the additional margins of 7, 5, and 2 mm to the A–P, S–I, and M–L dimension should adequately cover the extreme chest wall movement in 95 % of the patients. Nevertheless, for clinical practice, margin adding should be individualized as much as possible. Also adding additional margins for setup error might be different from institute to institute.

## Conclusions

This study show the maximal movement of the chest wall is in the anterior–posterior axis with is about 5.5–7.18 mm to coverage 95 % of the patients so additional margin of >7.1 mm especially in anterior–posterior axis is required to ensure the clinical target volume coverage from data of this study. But this study does not account for the error in interfraction patients’ setup. So, the additional setup margin should be considered in each setting.
